# Interocular Axial Length Difference and Treatment Outcomes of Anisometropic Amblyopia

**DOI:** 10.18502/jovr.v17i2.10791

**Published:** 2022-04-29

**Authors:** Monireh Ghasempour, Masoud Khorrami-Nejad, Aidin Safvati, Babak Masoomian

**Affiliations:** ^1^Department of Neuroscience, Faculty of Advanced Technologies in Medicine, Iran University of Medical Sciences, Tehran, Iran; ^2^Optometry Department, School of Rehabilitation, Tehran University of Medical Sciences, Tehran, Iran; ^3^Translational Ophthalmology Research Center, Farabi Eye Hospital, Tehran University of Medical Sciences, Tehran, Iran; ^4^School of Optometry and Vision Science, University of New South Wales, Sydney, Australia

**Keywords:** Anisometropic Amblyopia, Axial Length, Refractive Error

## Abstract

**Purpose:**

To evaluate the effect of interocular axial length (AL) difference on outcomes of treatment for anisometropic amblyopia in comparison with normal participants.

**Methods:**

In this historical cohort study, 83 patients with anisometropic amblyopia were divided into two age groups, 70 children (mean, 7.86 ± 1.56 and range, 5–15 years) and 13 adults (mean, 26.46 ± 10.87 and range, 16–45 years). The control group consisted of 43 non-amblyopic children and 17 non-amblyopic adults. Treatment outcomes after a period of one year were defined as successful or unsuccessful when posttreatment amblyopic corrected distance visual acuity (CDVA) was reported as ≤ 0.9 versus CDVA ≤ 0.8, respectively. AL was measured using a Lenstar LS900 (Haag-Streit AG, Switzerland).

**Results:**

Fifty-nine patients showed satisfactory treatment outcomes (55 children and 4 adults), while unsuccessful treatment outcomes were observed in 24 patients (15 children and 9 adults). The mean of amblyopia treatment duration was 1.24 ± 0.76 years. The mean of interocular AL difference in all patients, control, successful and unsuccessful treatment outcome groups were 0.49 ± 0.70mm (range, 0.00–3.89 mm), 0.12 ± 0.07 mm (range, 0.02–0.41), 0.33 ± 0.23 mm (range, 0.00–0.99 mm), and 1.81 ± 0.80 mm (range, 1.14–3.89 mm), respectively. In both age groups, the mean of interocular AL difference in patients with unsuccessful treatment outcomes was greater than those with successful treatment outcomes and that of the control group (*P* < 0.001).

**Conclusion:**

The results of this study suggest that the outcome of anisometropic amblyopia treatment may depend on the interocular AL difference.

##  INTRODUCTION

Anisometropic amblyopia is one of the most common types of amblyopia,^[1,2,3]^ which is secondary to differences in refractive errors between the two eyes.^[4,5]^ Amblyopia is a type of incomplete development of the visual system occurring in early childhood.^[5,6]^ It is one of the main causes of lifelong visual impairment^[3]^ and is the most common cause of vision loss in childhood affecting 3.5% of children.^[7]^ If amblyopia is not diagnosed or treated in due course, there will be a higher risk of vision impairment by conditions such as macular degeneration that may potentially occur in the non-amblyopic eye. Based on a Danish study, about 1.2% of patients with amblyopia will eventually suffer from severe visual impairment.^[10]^

One of the main causes of amblyopia is uncorrected refractive error, which is hard to detect when compared to amblyopia caused by strabismus.^[5,7,11]^ In fact, the initial factor causing anisometropic amblyopia (monocular blur) is the uncorrected refractive error. It leads to the inequality of image size between the two eyes. The blurriness of the image encumbers proper stimulation of the visual system and thus functions as an underlying cause of amblyopia. ${}^{\left[4,5\right]}$ Although exceptions exist, various studies have reported that higher amounts of anisometropia may result in amblyopia with increased severity.^[12,13]^

Despite the body of knowledge existing about the major risk factors such as the age of onset, the severity of anisometropia and the type of treatment, treatment of anisometropic amblyopia is reportedly unsuccessful in 65% to 94% of patients.^[5]^ Therefore, one could postulate that unknown risk factors may be involved, warranting further investigation into this area.

Anisometropia is defined as a difference in refractive error of ≥ 1.00 diopter (D) between the two eyes.^[6,14]^ Various studies have shown that with an increase in anisometropia, a higher difference in the axial length (AL) between the two eyes may occur as well.^[15,16,17]^ The severity of amblyopia is also one of the major risk factors for the failure of anisometropic amblyopia treatment.^[7,11,18,19]^

It seems a combination of these risk factors may influence the results of anisometropic amblyopia treatment, but it is not clear which factor is the most effective.

The aim of this study was to evaluate the effect of the interocular AL difference of each individual on the outcomes of anisometropic amblyopia treatment.

##  METHODS

This historical cohort study was conducted at Najafzadeh Eye Clinic, Karaj, Iran from September 2016 to October 2018. In this study, data were collected observationally. The study was performed in accordance with the tenets of the Declaration of Helsinki and approved by the ethics committee of our clinic.

Eighty-three patients with anisometropic amblyopia with no congenital anomalies, organic lesions, fixation disorders, strabismus, or glaucoma, were included in this study. They were divided into two age groups, 5–15 years (*n* = 70) and 16–45 years (*n* = 13). We also included a control group consisting of 43 non-amblyopic children and 17 non-amblyopic adults. Anisometropic amblyopia was defined as a difference of at least two lines in corrected distance visual acuity (CDVA) between the two eyes with the CDVA being equal to or worse than 20/30 due to uncorrected refractive error also with a difference of > 1.50 D in spherical refractive error between the two eyes.^[2,17]^

With each age group we had two treatment outcomes: (1) a successful treatment outcome (group A); whereby satisfactory CDVA ( ≥ 0.90) was achieved following treatment of amblyopia using corrective glasses alone or with occlusion therapy and (2) an unsuccessful treatment outcome with residual amblyopia (group B), defined as CDVA ≤ 0.8 after at least one year of treatment. Control, non-amblyopic participants, were those who had CDVA ≥ 1.00 and at least ± 0.50 D anisometropia.

Routine ophthalmic examinations detailed below were performed for all participants. CDVA was performed using an E-Snellen chart at a distance of 6 m, and the findings were presented in decimals. Alternate cover tests, versions and duction tests were performed to rule out strabismus and extraocular muscle movement limitations. A slit‐lamp (SL-202Ⓡ, Shin-Nippon, Japan) was used to perform biomicroscopy and funduscopy for all amblyopic and normal participants.

Dry and cyclo refraction were measured by a Nidek ARK-710A auto keratorefractometer (Nidek Co. Ltd, Gamagori, Japan) and confirmed by retinoscopy (HEINE Optotechnic, Hersching, Germany). All participants underwent cyclorefraction by administering cyclopentolate 1% (two drops, 5 min apart, followed by refraction 30 min after the last drop).

Amblyopia treatment in all patients was started with at least three months of full-time wearing of corrective glasses. As described by the Pediatric Eye Disease Investigator Group (PEDIG) studies, supplementary amblyopia therapies were provided at the end of three months.^[20]^ In patients with unsuccessful treatment, the amblyopia treatment program was continued for at least one year. The mean duration of amblyopia treatment was also calculated. Depending on the severity of amblyopia, treatment was continued until the resolution of the amblyopia. Upon discontinuation of treatment, patients were followed-up for at least six months. In the last visit of patients, the AL of both eyes was measured (Lenstar LS900, Haag-Streit AG, Switzerland), and the average of five measurements was recorded.

Statistical analysis was performed using SPSS v22 software for Windows (IBM Inc., Armonk, New York, NY, USA). * P*-values < 0.05 were recorded as statistically significant. The Shapiro–Wilk test was used to evaluate the normal distribution of the interocular AL difference, and the Kruskal–Wallis test was performed to compare the interocular AL differences between the three groups (successful treatment outcome, unsuccessful treatment outcome, and control group).

**Table 1:** The comparison of the pretreatment of corrected distance visual acuity (LogMAR) between children and adult groups, based on amblyopia treatment outcomes in anisometropic amblyopic patients.


**Amblyopia treatment outcomes**	**Age group (yr)**	**Number**	**Minimum**	**Maximum**	**Mean ± SD**	**Mean standard error**	***P-*** **value**
Good treatment outcome	5–15	55	0.18	1.00	0.43 ± 0.19	0.02	0.152
		16–45	4	0.18	0.48	0.28 ± 0.14	0.07	
Failure of successfully treatment outcome	5–15	15	0.40	1.30	0.78 ± 0.35	0.09	0.499
		16–45	9	0.40	1.30	0.68 ± 0.29	0.09	
	
	
SD, standard deviation; yr, year							

**Table 2:** Comparison of the mean anisometropia (diopter) among children and adult groups based on amblyopia treatment outcomes.


	**Age group (yr)**	**Number**	**Minimum**	**Maximum**	**Mean ± SD**	**Mean standard error**	***P*** **-value**
Control group	5–15	43	0.50	1.50	0.86 ± 0.25	0.04	0.279
		16–45	17	0.50	1.75	0.92 ± 0.33	0.08	
Good treatment outcome	5–15	55	1.75	3.00	2.01 ± 0.33	0.05	0.536
		16–45	4	1.75	3.25	2.25 ± 0.68	0.34	
Failure of successful treatment outcome	5–15	15	3.50	11.50	5.57 ± 2.43	0.63	0.329
		16–45	9	2.25	8.25	4.61 ± 2.19	0.73	
	
	
SD, standard deviation; yr, year							

**Table 3:** Comparison of the mean of the interocular axial length difference (mm) between the children and adult group based on amblyopia treatment outcomes.


	**Age group (yr)**	**Number**	**Minimum**	**Maximum**	**Mean ± SD**	**Mean standard error**	***P-*** **value**
Control group	5–15	43	0.02	0.41	0.12 ± 0.08	0.01	*P *= 0.458
		16–45	17	0.03	0.28	0.13 ± 0.06	0.02	
Good treatment outcome	5–15	55	0.00	0.99	0.34 ± 0.25	0.03	*P *= 0.407
		16–45	4	0.05	0.38	0.22 ± 0.14	0.07	
Failure of successful treatment outcome	5–15	15	1.14	3.89	1.90 ± 0.91	0.24	*P *= 0.788
		16–45	9	1.16	3.00	1.69 ± 0.61	0.20	
	
	
SD, standard deviation; yr, year							

**Figure 1:**
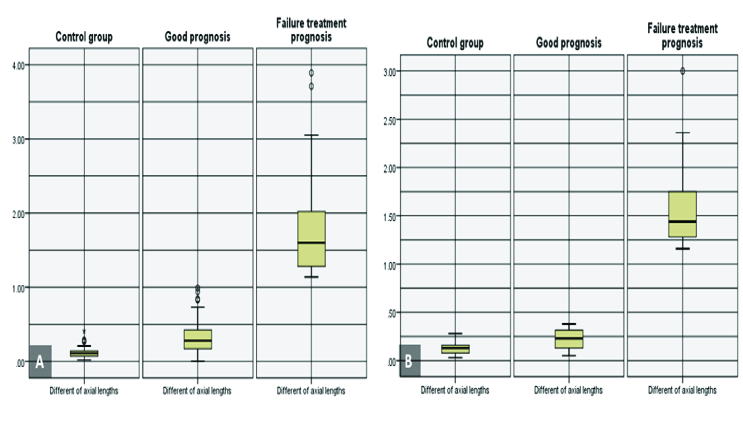
The box plot distribution of the interocular axial length difference (mm), categorized based on amblyopia treatment outcomes in the children (A) and adults (B) groups.

**Figure 2:**
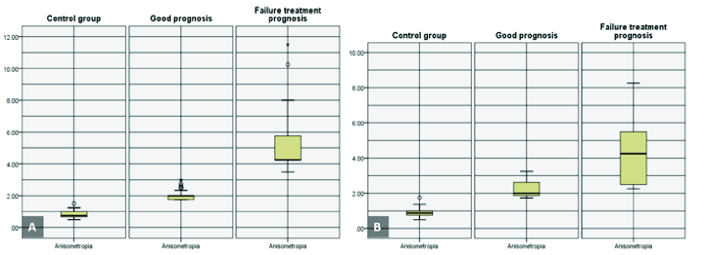
The box plot distribution of the anisometropia (diopter), categorized based on amblyopia treatment outcomes in the children (A) and adult (B) groups.

##  RESULTS

Eighty-three patients with anisometropic amblyopia were tested for this study (35 females and 48 males) with a mean age of 10.77 ± 8.10 years (range, 5–45). They were divided into two age groups, 5–15 years (group A, *n* = 70, mean 7.86 ± 1.56) and 16–45 years (group B, *n* = 13, mean 26.46 ± 10.87). Additionally, 60 participants were selected as a control group consisting of non-amblyopic anisometropic participants (43 children aged 5–15 years with a mean age of 10.42 ± 2.95 years and 17 adults aged 16–45 years with a mean age of 22.47 ± 4.73 years). In the children's group (group A), the time of diagnosis and first treatment was between the ages of 4 and 6 years, while in the adult group, it was at 15 years old and above. For all patients, the mean duration of amblyopia treatment was 1.24 ± 0.76 years, with a maximum of 2.50 years. This treatment period included at least three-months of full-time refractive correction (without any other active or passive modes of treatment for amblyopia). Hours of patching for mild, moderate, and severe amblyopia were 2, 4, and 6 hr, respectively. All amblyopic patients had refractive anisometropic amblyopia. Amblyopia was successfully treated in 59 (71.1%) patients (55 children and 4 adults), while 24 (28.9%) patients demonstrated an unsuccessful treatment outcome with residual amblyopia (15 children and 9 adults).

The comparison of the pretreatment CDVA (LogMAR) between children and adult groups, categorized based on amblyopia treatment outcomes in anisometropic amblyopic patients, are shown in Table 1. The mean of pretreatment CDVA in the children and the adult group was 0.51 ± 0.28 (range, 0.18–1.30) and 0.56 ± 0.31 (range, 0.18–1.30), respectively. After the treatment of amblyopia, the mean of CDVA in the children and the adult group was 0.12 ± 0.29 (range, 0.00–1.30) and 0.30 ± 0.37 (range, 0.00–1.30), respectively.

The mean of anisometropia in all anisometropic amblyopia patients, children and adult groups were 2.94 ± 1.94 D (range, 1.75–11.50), 2.77 ± 1.85 D (range, 1.75–11.50), and 3.88 ± 2.14 D (range, 1.75–8.25), respectively. Table 2 shows the comparison of the mean of anisometropia between the children and the adult groups, categorized by the treatment outcome.

The mean of the interocular AL differences in all patients was 0.63 ± 0.78 mm. Figures 1 and 2 show the box plot distribution of the interocular AL differences and amount of anisometropia, categorized by treatment outcomes in the children and the adult groups. As shown in Figure 1, the interocular AL difference in the unsuccessful treatment outcome group was > 1 mm (in both age groups).

The mean of the interocular AL difference was significantly larger in the anisometropic amblyopia group when compared to the control group (*P* < 0.001). Also, there was a significant difference between the control group, the unsuccessful and successful treatment outcome groups (*P* < 0.001). In both age groups, the mean of the interocular AL difference in the unsuccessful treatment outcome group was significantly greater than the successful treatment outcome group (*P* < 0.001).

In the children group, when a successful treatment outcome was achieved, the mean AL was larger than the control group (*z* = –5.539; *P* < 0.001; 2-tailed); however, in the adult group this difference was not statistically significant (*t* = –1.360; df = 3.323; *P* = 0.259). Table 3 shows the comparison of the mean of the interocular AL difference between children and adult groups as categorized by treatment outcome.

##  DISCUSSION

The findings of this study indicate that the interocular AL difference in patients with unsuccessful treatment outcomes was significantly larger than patients with successful treatment outcomes and those of the control group. Recent studies have indicated the existence of a significant correlation between anisometropia and the interocular AL difference in anisometropic amblyopia patients.^[13,15,17,21,22]^

Studies suggest that amblyopia cannot be treated beyond a critical age because of the lack of adequate plasticity in the adult brain.^[23]^ In this study, amblyopia was treated successfully in four adults. The findings of this study indicate that even in children, amblyopia may not be treated successfully in all patients. Bonacorci et al reported that due to the flexibility of the visual system, the results of anisometropic amblyopic treatment could vary depending on the age of treatment.^[23]^ Meanwhile, Lia et al have not mentioned age as a main factor in treatment outcomes.^[24]^ We report that the mean of the interocular AL difference was larger when treatment was unsuccessful (compared to the successful treatment group and the controls). This finding was consistent for both the children and the adult group. In both age groups, it seems that the interocular AL difference may assist in predicting the outcomes of the amblyopia treatment.

In our study, the interocular AL difference was larger in the group with unsuccessful treatment outcomes when compared to the group with successful treatment and the controls. The higher interocular AL difference in the group with unsuccessful treatment outcomes confirms that the hypermetropic amblyopic eye had an AL that was shorter than the non-amblyopic eye. Lempert et al in a study involving 927 aniso-hypermetropic amblyopic patients aimed at examining the AL to the optic nerve disk area ratio (AL/DA).^[25]^ According to the report, the area of the optic nerve disc in the amblyopic eyes was significantly smaller than that of the non-amblyopic eyes. Also, the size of the optic nerve disc and AL in the more hypermetropic eye was smaller than the optic disc and AL of the non-amblyopic eye. Furthermore, in a study on monkeys, Swadlow et al have reported that the diameter of axons with the highest transmission speed was 20 microns. In comparison, the axons with the slowest speed of transmission had a diameter of about 0.1 micron.^[26]^ Based on these two studies, it can be postulated that the speed of transmission of neural impulses may be linked to the diameter of axons. It can also be suggested that perhaps interocular AL and optic disc size differences lead to some sort of interference in the speed of transmission of retinal images between the retina and the visual cortex, thus creating an abnormal development of the visual cortex of the amblyopic eyes.

Our results suggest that in anisometropic amblyopes who achieve better treatment outcomes as well as in controls, AL shows smaller differences between the two eyes. Interestingly, this finding was similar across the two tested age groups. Based on the results of this study, the difference of AL between the two eyes of each individual may assist with the prediction of treatment outcomes in anisometropic amblyopic patients. Although our study has successfully determined the existing relationship between interocular AL difference and treatment outcomes for anisometropic amblyopia, further studies using larger sample sizes are warranted to expand our knowledge on the topic.

##  Financial Support and Sponsorship

Nil.

##  Conflicts of Interest

None declared.
